# Supplementary material for: Automated evaluation systems to enhance exam quality and reduce test anxiety
Source: PeerJ Comput Sci. 2025 Feb 25;11:e2666. doi: 10.7717/peerj-cs.2666 (PMC11888855; doi:10.7717/peerj-cs.2666)
Supplement: Supplemental Information 1 [file peerj-cs-11-2666-s001.pdf]

Evaluate the performance of the proposed system

- Is system usability
  - poor
  - fair
  - good
  - very good
  - excellent

- Is system effectiveness
  - poor
  - fair
  - good
  - very good
  - excellent

- Is system short response time
  - poor
  - fair
  - good
  - very good
  - excellent

- Is system clarity
  - poor
  - fair
  - good
  - very good
  - excellent

- Is system applicability
  - poor
  - fair
  - good
  - very good
  - excellent

NOTES: (1= Poor, 2= Fair, 3= Good, 4= Very Good, 5= Excellent)
