# Supplementary material for: Automated evaluation systems to enhance exam quality and reduce test anxiety
Source: PeerJ Comput Sci. 2025 Feb 25;11:e2666. doi: 10.7717/peerj-cs.2666 (PMC11888855; doi:10.7717/peerj-cs.2666)
Supplement: Supplemental Information 4 [file peerj-cs-11-2666-s004.docx]

**A scale for students' academic stress and its relationship with the quality of the exam paper**

**1 . Statements about academic stress:**

I_1 : I feel tense and very anxious before exams.

I_2 : I find it difficult to sleep the night before an exam.

I_3 : I feel tense and under psychological pressure when thinking about upcoming exams.

I_4 : I have difficulty concentrating while studying for exams due to anxiety.

I_5 : I frequently worry about my performance in exams.

I_6 : I feel exhausted and stressed during the exam period.

I_7 : I suffer from headaches or stomach pains before or during exams.

I_8 : I constantly think about exams and the grades I will get.

**2. Statements about the quality of the exam paper:**

T_1 : I feel that the questions on the exam paper are clear and understandable.

T_2 : I believe that the available time to answer the questions in the exam is sufficient.

T_3 : I feel that the exam paper fairly assesses my knowledge of the subject.

T_4 : I feel that the exam's format and arrangement of questions help me perform better.

T_5 : I feel that the questions reflect what has been covered in lectures and lessons.

T_6: There are clear instructions on the exam paper on how to answer.

T_7 : I feel that the exam paper's format and the presence of basic course data help me perform well.

**3. Statements linking the quality of the exam to academic stress:**

F_1 : When the exam questions are clear and well-organized, I feel less anxious.

F_2 : If the exam includes unclear or misleading questions, my feeling of academic stress increases.

F_3 : I feel that my chances of success increase when the exam paper is organized and balanced in difficulty.

F_4 : Anxiety about the exam decreases when I know that the questions will be fair and reflect the material that was taught.
